# Multiple Introductions of SARS-CoV-2 Alpha and Delta Variants into White-Tailed Deer in Pennsylvania

**DOI:** 10.1128/mbio.02101-22

**Published:** 2022-08-24

**Authors:** Andrew D. Marques, Scott Sherrill-Mix, John K. Everett, Hriju Adhikari, Shantan Reddy, Julie C. Ellis, Haley Zeliff, Sabrina S. Greening, Carolyn C. Cannuscio, Katherine M. Strelau, Ronald G. Collman, Brendan J. Kelly, Kyle G. Rodino, Frederic D. Bushman, Roderick B. Gagne, Eman Anis

**Affiliations:** a Department of Microbiology, Perelman School of Medicine, University of Pennsylvania, Philadelphia, Pennsylvania, USA; b Department of Pathobiology, Wildlife Futures Program, University of Pennsylvania School of Veterinary Medicine, New Bolton Center, Kennett Square, Pennsylvania, USA; c Department of Pathobiology, University of Pennsylvania School of Veterinary Medicine, New Bolton Center, Kennett Square, Pennsylvania, USA; d Leonard Davis Institute of Health Economics, University of Pennsylvania, Philadelphia, Pennsylvania, USA; e Department of Family Medicine and Community Health, University of Pennsylvania, Philadelphia, Pennsylvania, USA; f Pulmonary, Allergy and Critical Care Division; Department of Medicine; University of Pennsylvania Perelman School of Medicine; Philadelphia, Pennsylvania, USA; g Division of Infectious Diseases; Department of Medicine & Department of Biostatistics, Epidemiology, and Informatics; Perelman School of Medicine, University of Pennsylvania, Philadelphia, Pennsylvania, USA; h Department of Pathology and Laboratory Medicine, Perelman School of Medicine, University of Pennsylvania, Philadelphia, Pennsylvania, USA; University of California, Davis

**Keywords:** SARS-CoV-2, coronavirus, white-tail deer, *Odocoileus virginianus*, animal reservoir, zoonosis

## Abstract

The SARS-CoV-2 pandemic began by viral spillover from animals to humans; today multiple animal species are known to be susceptible to infection. White-tailed deer, Odocoileus virginianus, are infected in North America at substantial levels, and genomic data suggests that a variant in deer may have spilled back to humans. Here, we characterize SARS-CoV-2 in deer from Pennsylvania (PA) sampled during fall and winter 2021. Of 123 nasal swab samples analyzed by RT-qPCR, 20 (16.3%) were positive for SARS-CoV-2. Seven whole genome sequences were obtained, together with six more partial spike gene sequences. These annotated as alpha and delta variants, the first reported observations of these lineages in deer, documenting multiple new jumps from humans to deer. The alpha lineage persisted in deer after its displacement by delta in humans, and deer-derived alpha variants diverged significantly from those in humans, consistent with a distinctive evolutionary trajectory in deer.

## INTRODUCTION

Multiple spillovers of coronaviruses from animals to humans have founded epidemics in humans. Examples include spillover of SARS from bats to humans, likely with civets as an intermediate host, and spillover of middle east respiratory syndrome (MERS) from bats to camels to humans (1–3). For SARS-CoV-2, the epidemic likely began with spillover from *Rhinolophus* bats to humans, possibly via an intermediate host (4, 5).

SARS-CoV-2 infection has further “spilled back” from humans into numerous animal species (6–10), creating the risk of formation of new animal reservoirs. Examples of animals known to be infectible with SARS-CoV-2 include great apes, mice, cats, dogs, deer, mink, and hamsters (6, 8, 11–16). Infection of mink farms has been widely reported, and tracking via analysis of genome sequences suggests that a mink lineage has spilled back into humans (6).

The first indications of the establishment of a potential wildlife reservoir of concern have been observed in white-tailed deer, Odocoileus virginianus. White-tailed deer appear to have been widely infected in North America (11, 17, 18). Comparison of genomic sequences from the Alberta region suggest that the virus evolved a novel lineage during transmission in deer, and that this spilled back on at least one occasion to a human from the same region (19, 20).

Here, we report a study of infection and evolution of SARS-CoV-2 in the Pennsylvania region. White-tailed deer were sampled throughout Pennsylvania from 10/2/2021 to 12/27/2021 with the goal of characterizing this potential new animal reservoir. Samples were tested for the presence of SARS-CoV-2 by qPCR, and the viral lineages present assessed using viral whole genome sequencing after multiplex PCR. For samples with lower RNA amounts, sequencing was carried out on a single nested PCR amplicon encoding the spike receptor binding domain, which allows variant profiling. Results report the nature of this emerging reservoir in a heavily populated state.

## RESULTS

### Tracking SARS-CoV-2 in white-tailed deer using RT-qPCR.

SARS-CoV-2 was detected by RT-qPCR in nasal swabs from 20 of 123 wild white-tailed deer sampled (16.3%; 95% confidence interval (CI): 10.4, 24.2) (described in Table S1 and S2). There was no significant difference in infection frequency associated with the cause of death (roadkill 7/24, and hunter-harvested 12/79; *P*-value = 0.14). There was no significant difference in infection rates between sexes (females 6/43, males 14/60; *P*-value = 0.46) and age groups (fawn 1/18, yearling 1/22, and adult 17/81; *P*-value = 0.093). No information was available on possible symptoms or disease for the animals sampled.

10.1128/mbio.02101-22.1 TABLE S1 SARS-CoV-2 prevalence estimates stratified by sex, age, sampling region, and cause of death. Download Table S1, PDF file, 0.4 MB. Copyright © 2022 Marques et al. 2022 Marques et al. https://creativecommons.org/licenses/by/4.0/ This content is distributed under the terms of the Creative Commons Attribution 4.0 International license.

10.1128/mbio.02101-22.2 TABLE S2 Results of qPCR and sequencing analysis. Download Table S2, XLSX file, 0.03 MB. Copyright © 2022 Marques et al. 2022 Marques et al. https://creativecommons.org/licenses/by/4.0/ This content is distributed under the terms of the Creative Commons Attribution 4.0 International license.

**FIG 1:**
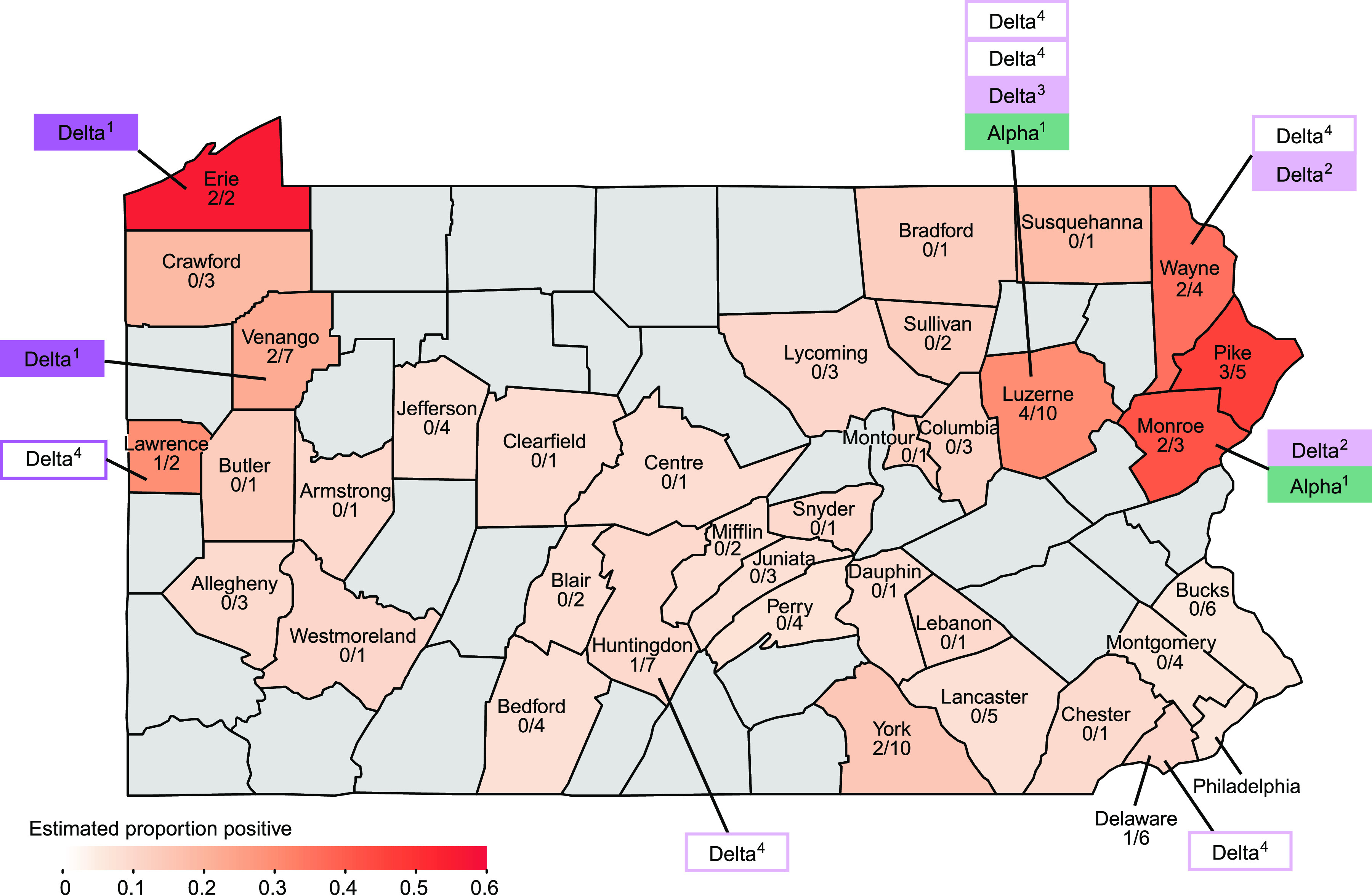
Map of Pennsylvania (PA), showing sampling sites and locations of SARS-CoV-2 positive deer. The counties comprising PA are outlined. The estimated proportion of positive samples is shown by the white-to-red color scale; counties that were not sampled are shown in gray. Total sample numbers and the number positive are written on each county sampled. The deer samples sequenced were assigned to variants as indicated by the rectangles outside the map; variant type is color-coded (teal for alpha/B1.1.7, purple/pink for delta/AY.#). The open boxes indicate sequences were available for the spike-only.

Virus positive deer were identified in 10 of the 31 Pennsylvania counties surveyed (Fig. 1). We used a Bayesian hierarchical binomial model to estimate positivity and predicted an average county had 9.9% positivity (95% CrI: 1.7-22%). Pike and Monroe counties were associated with higher apparent prevalence (Monroe: 7.5x higher odds (1.0-110x) of positivity; Pike: 9.3x higher odds [1.3-110x]). Grouping by region also showed a significant difference in infection rate (*P*-value = 0.0047), with the northeast region of PA showing a higher proportion of positive deer than the southeast region (Fisher’s exact test with false discovery rate (FDR) correction; *P*-value = 0.017).

### Assessing SARS-CoV-2 in white-tailed deer using viral whole genome sequencing.

Eight samples had viral RNA concentrations that in human specimens typically yield successful whole genome sequencing (cycle threshold values from RT-qPCR less than 30), and high-quality genome sequences were recovered from 7 (Table S2). To confirm sample provenance, the non-viral sequence reads were analyzed for the deer samples and for human samples that were sequenced in parallel. All deer samples yielded reads mapping to the deer genome, and few or none mapping to the human genome. In contrast, reads from human samples overwhelmingly mapped to the human genome (Fig. S1), confirming the host species origin of our samples as deer.

10.1128/mbio.02101-22.8 FIG S1 Checking sample tracking by analysis of nonviral sequences. Sequence reads from each SARS-CoV-2 sample were aligned to the white-tailed deer (GCF_002102435.1) and human (GCF_000001405.39) genomes, and nonviral reads enumerated. The numbers of deer and human reads are shown for each deer sample, denoted by the laboratory accession number VSP###. Results for 140 human samples, sequenced in the same sample batches, are shown to the far left. The proportion of reads aligning to the deer genome are shown in green, the average fraction aligning to the human genome is shown in orange. Download FIG S1, PDF file, 0.1 MB. Copyright © 2022 Marques et al. 2022 Marques et al. https://creativecommons.org/licenses/by/4.0/ This content is distributed under the terms of the Creative Commons Attribution 4.0 International license.

Samples with lower SARS-CoV-2 RNA concentrations (Ct ≥30) were amplified using a single nested PCR amplicon targeting the spike coding region and sequenced, yielding spike sequences from six additional samples. Spike sequences were then used for lineage assignment (21) (Table S2).

### Characteristics of SARS-CoV-2 genome sequences from deer in Pennsylvania.

**FIG 2:**
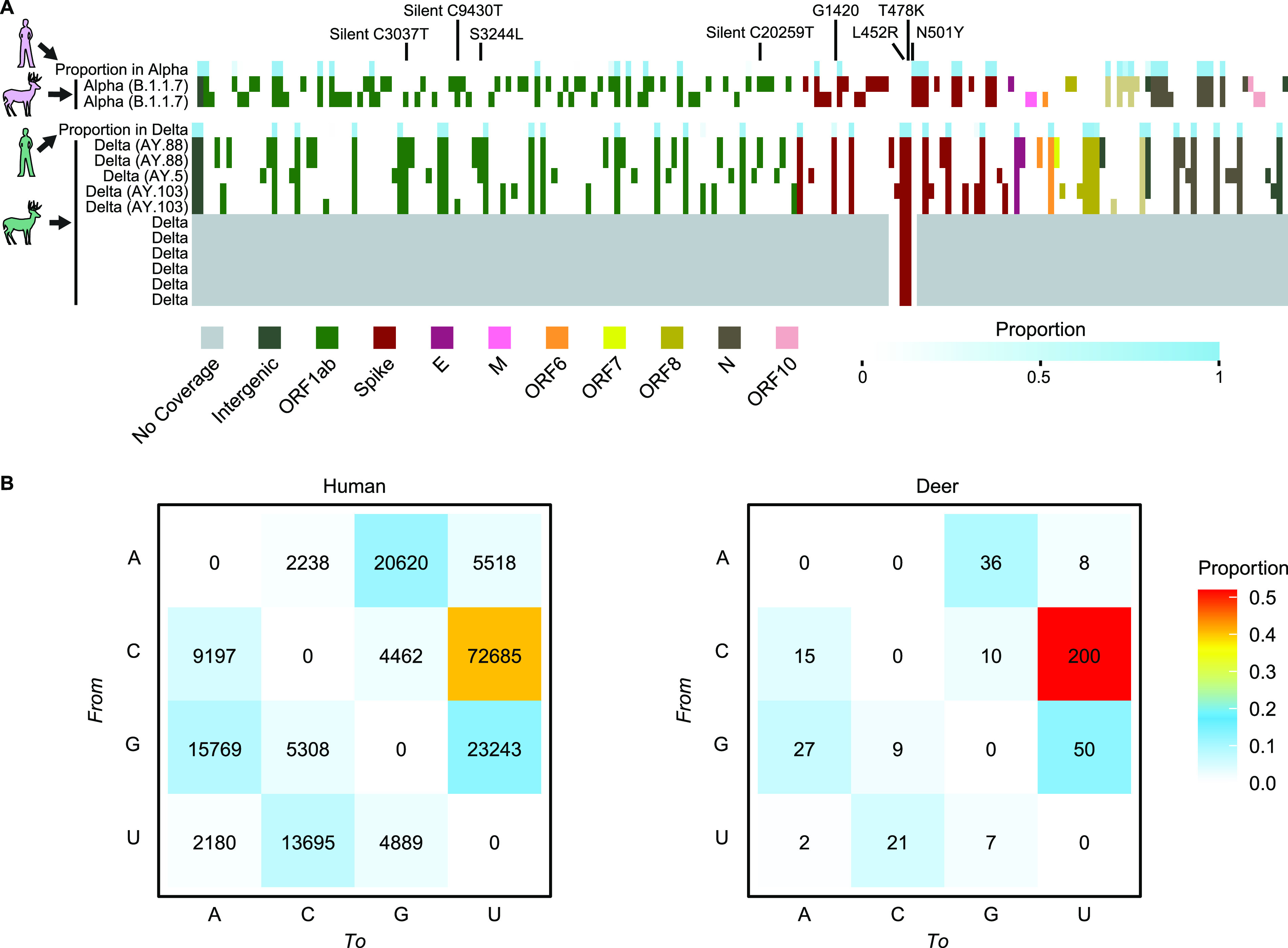
Base substitutions detected in sequences of deer from Pennsylvania. In (A) the proportions in humans sampled in the Delaware Valley is shown in blue along the top for alpha (upper) and delta (lower). Substitutions relative to the Wuhan strain references are shown by the colored boxes. The proportions in humans are shown by the blue shading in each box. Genes sampled are shown by the color code indicated along the bottom. Gray indicates lack of sampling. The bottom six rows of the delta samples indicate the spike-only amplicon sequences. (B) Types of base substitutions away from the Wuhan reference detected in humans (right) and deer (left). The proportions are shown by the color code at the left.

Deer whole genome sequences from Pennsylvania showed divergences relative to the original Wuhan SARS-CoV-2 strain and previously reported human sequences, and were distinct from prior sequences reported from deer (Fig. 2A). A full list of deer substitutions is in Tables S3. The complete deer genome sequences reported here annotated as either alpha or delta variants (22), the first reported identification of these lineages in wild white-tailed deer. Previously the alpha variant was shown to be able to infect white-tailed deer that were experimentally inoculated (18). All the spike-only sequences harbored polymorphisms that place them in the delta lineage and were inconsistent with other common lineages.

10.1128/mbio.02101-22.3 TABLE S3 Deer SARS-CoV-2 substitutions from sequences in this study. Download Table S3, XLSX file, 0.02 MB. Copyright © 2022 Marques et al. 2022 Marques et al. https://creativecommons.org/licenses/by/4.0/ This content is distributed under the terms of the Creative Commons Attribution 4.0 International license.

The types of base changes associated with substitutions were not uniform. Changes from C in the Wuhan reference to U were found to be the most frequent (Fig. 2B). The frequency of C to U was even higher in deer than in humans (Pearson’s Chi-squared test *P* = 0.031, followed by a *post hoc* analysis based on residuals yielding *P*-value = 0.0010 for C to U substitutions), consistent with a previous study (20), and suggestive of host-specific mutation rates.

To identify any deer-specific sequence polymorphisms, the alpha and delta SARS-CoV-2 sequences from deer determined here were compared to viral genomes from humans and deer reported previously. All deer genome sequences available from GISAID (*n* = 108) were downloaded (Table S4 and S5), corresponding to samples acquired from 9/28/2020 to 2/25/2021 in Iowa and Ohio and dominated by early pandemic lineages including B.1.2 and B.1.311 (11, 17). Local human isolates were derived from our SARS-CoV-2 sequencing program monitoring the Delaware Valley, including Philadelphia, PA (23, 24). This revealed several substitutions that were highly enriched or invariant in deer isolates, but rare or absent in human isolates. These include 3 silent mutations in ORF1ab, C7303U, C9430U, and C20259U. Mutation C7303U was found in 86% of these PA deer and 29% of previously published deer, but in only 0.04% of humans in Delaware Valley and 0.09% of global SARS-CoV-2 genomes reported by NextStrain (data from 1/26/2022). Mutation c9430t was found in 43% of PA deer and 56% of previously published deer, but 0.35% of Delaware Valley and 0.46% of global human SARS-CoV-2 genomes. Mutation C20259U was found in 43% of PA deer and 19% of previously published deer, whereas it was absent in Delaware Valley human subjects and present in 0.12% of global genomes. The enrichment of these mutations suggests possible functional interaction with deer-specific factors, which could influence RNA synthesis, RNA folding, or protein binding.

10.1128/mbio.02101-22.4 TABLE S4 Single nucleotide polymorphisms from global whole genome sequencing of SARS-CoV-2 in deer. Download Table S4, XLSX file, 0.3 MB. Copyright © 2022 Marques et al. 2022 Marques et al. https://creativecommons.org/licenses/by/4.0/ This content is distributed under the terms of the Creative Commons Attribution 4.0 International license.

10.1128/mbio.02101-22.5 TABLE S5 Human and deer sequences used in analysis. Download Table S5, XLSX file, 0.3 MB. Copyright © 2022 Marques et al. 2022 Marques et al. https://creativecommons.org/licenses/by/4.0/ This content is distributed under the terms of the Creative Commons Attribution 4.0 International license.

**FIG 3:**
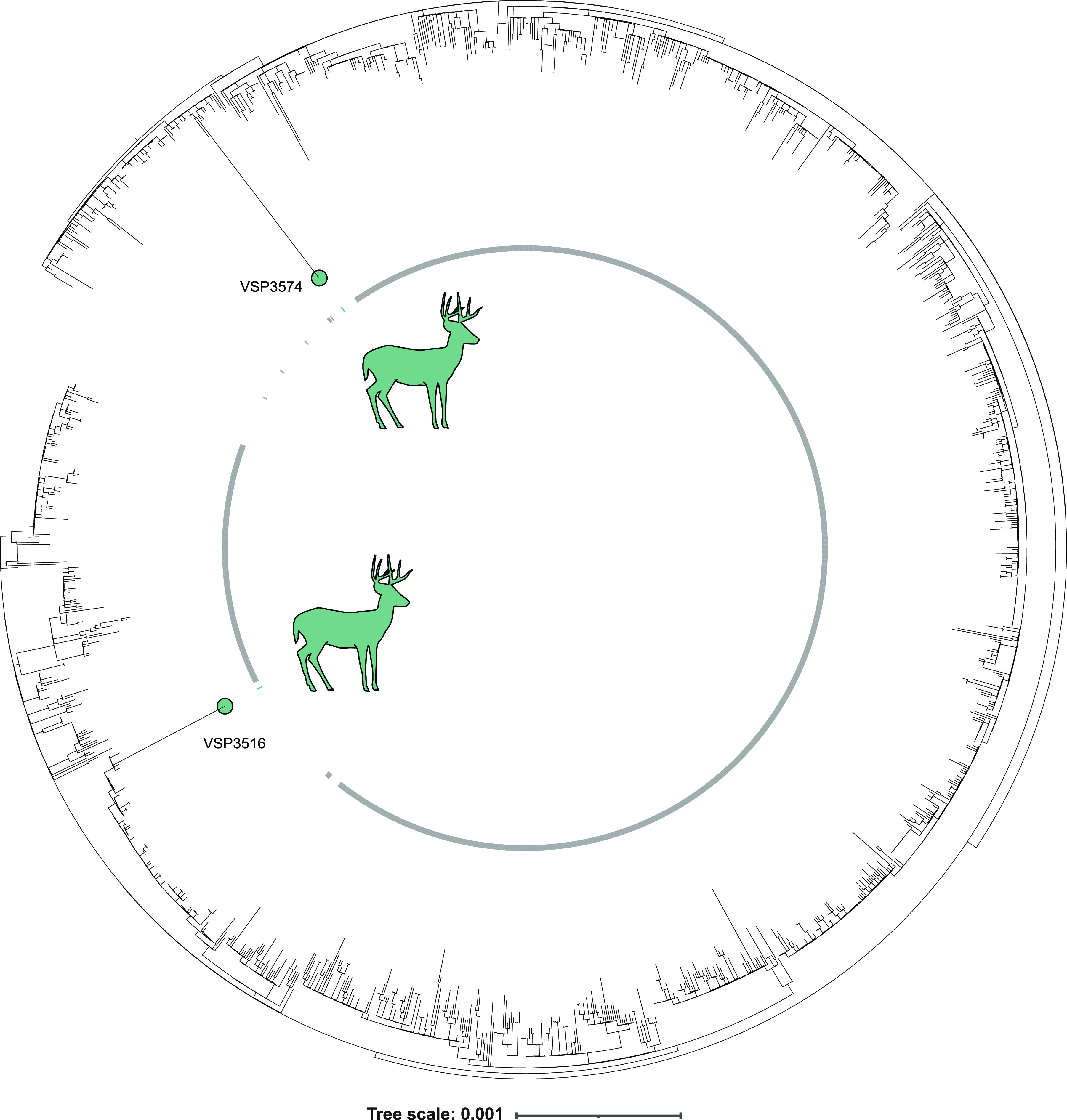
Analysis of alpha variant SARS-CoV-2 whole genome sequences from white-tailed deer, global nearest neighbor human-derived sequences, and Delaware Valley human-derived sequences. Phylogenetic analysis of alpha white-tailed deer-derived sequences, nearest global sequences, and contemporaneous human alpha lineage sequences from Delaware Valley, collected from 1/6/2021 to 11/16/2021(2 deer sequences, 200 global nearest neighbor human sequences, and 1239 contemporanious Delaware Valley human). Included in the contemporaneous Delaware Valley human samples are two examples of humans infected with the alpha variant during the delta wave as late as 10/17/2021 and 11/14/2021, although these samples are notably more similar to earlier human alpha sequences than the deer alpha sequences. Dear sequences are colored teal, contemporaneous Delaware Valley sequences are labeled gray, and global nearest neighbor sequences are uncolored.

**FIG 4:**
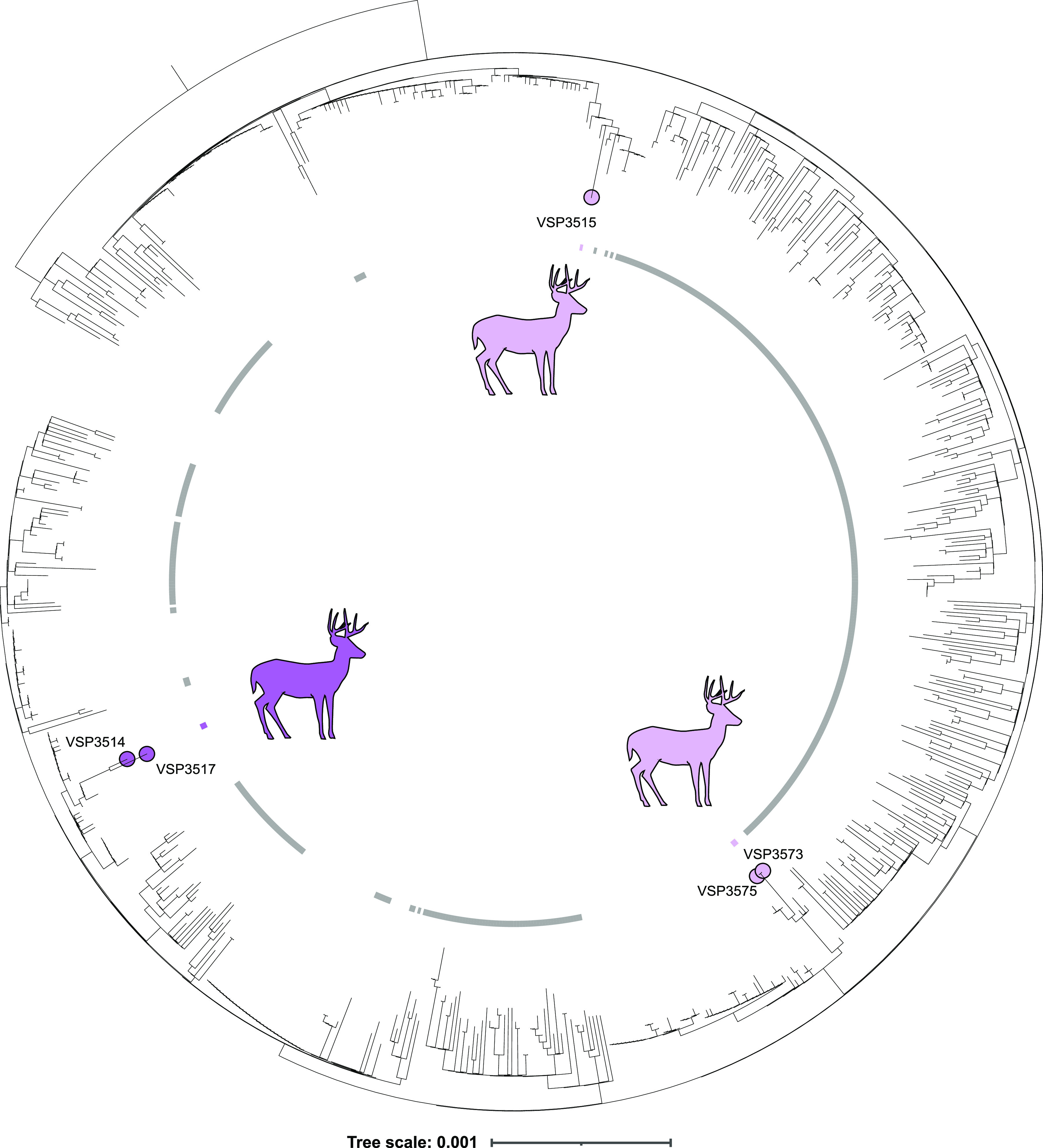
Phylogeny of delta white-tailed deer sequences, nearest global human sequences, and human delta lineage sequences from Delaware Valley, collected from 10/14/2021 to 11/28/2021 (spanning the period of genomes obtained from deer). Included are 5 deer, 300 global nearest neighbor human, and 440 Delaware Valley human sequences. Deer sequences are marked in purple, Delaware Valley human sequences are marked in gray, and global nearest neighbor sequences are unmarked. Time resolved trees for each spillover are in Fig. S2.

**FIG 5:**
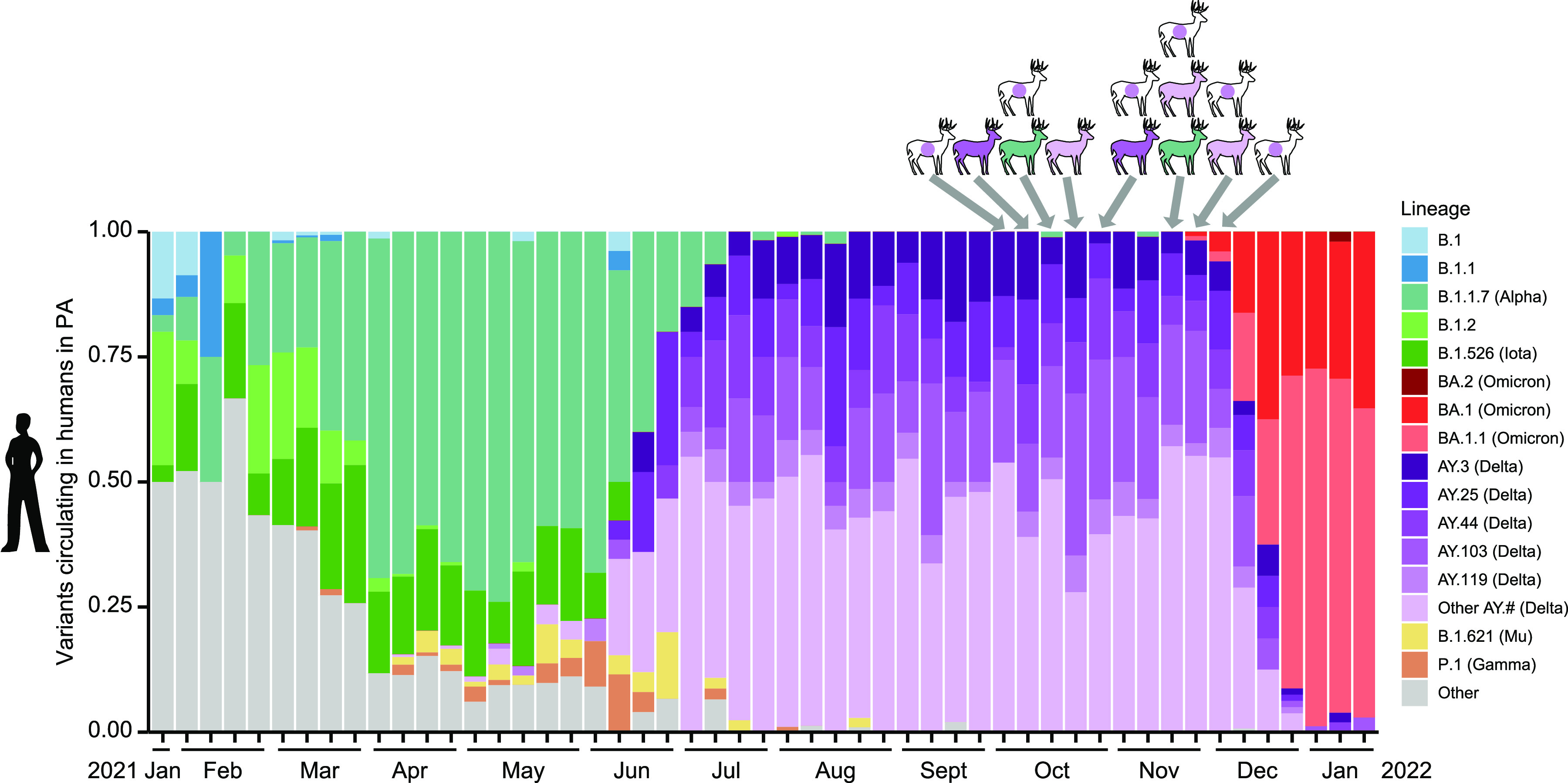
Longitudinal comparison of deer variants and human variants. The bar plots show the progression of SARS-CoV-2 variants detected by genome sequencing in humans in eastern Delaware Valley from 1/31/2021 to 1/3/2022. The variants are color-coded according to the key to the right. The variants from white-tailed deer sequences are shown at the top of the figure, with the arrows showing the times of sampling. The deer isolates are color-coded on the deer icons as in the key; purple dots on deer icons indicate sequences identified as delta lineage by spike amplicon sequences but without whole genome sequences to further identify clade.

To assess relationships among deer and human-derived SARS-CoV-2 sequences, we used NextClade for sequence alignment and IQ-TREE to construct a maximum-likelihood phylogenetic tree for viral sequences from alpha (Fig. 3) and delta (Fig. 4) variants, in each case comparing PA deer to their 100 nearest genetic neighbors from NCBI’s global human data set and contemporaneous human-derived sequences from the same variant in the Delaware Valley region. We also compared the deer lineages observed longitudinally to all contemporaneous human lineages sampled in PA (Fig. 5, and Table S5 and S6).

10.1128/mbio.02101-22.6 TABLE S6 Parameter tuning for time-scale Bayesian maximum clade credibility tree. Download Table S6, PDF file, 0.4 MB. Copyright © 2022 Marques et al. 2022 Marques et al. https://creativecommons.org/licenses/by/4.0/ This content is distributed under the terms of the Creative Commons Attribution 4.0 International license.

10.1128/mbio.02101-22.9 FIG S2 Time resolved phylogenies, comparing deer and human lineages. Deer isolates are compared to human isolates from an overlapping geographic region. Trees are shown for individual lineages when relatively divergent from other deer isolates, or for pairs of deer isolates when similar in sequence. Download FIG S2, PDF file, 0.3 MB. Copyright © 2022 Marques et al. 2022 Marques et al. https://creativecommons.org/licenses/by/4.0/ This content is distributed under the terms of the Creative Commons Attribution 4.0 International license.

The 2 alpha sequences sampled from deer came from adjoining counties in northeastern PA. The isolates differed by 45 substitutions and were widely separated on the phylogenetic tree. A parsimonious explanation is that the two alpha lineages were introduced independently into deer and then diversified during transmission within deer. Molecular clock analysis yielded data consistent with a model in which alpha jumped from humans to deer as early as March/April of 2021 (95% credible interval [CrI] for VSP3574: 3/10/2021 to 3/30/2021; and for VSP3516: 4/23/2021 to 5/4/2021), during the alpha wave, and persisted in deer up until the sampling time 6 to 7 months later in mid-November, when alpha was no longer circulating in the US human population (Fig. S2 and Table S5). The modeled estimates of the timing of spillover to deer have high uncertainties and provide only an early bound on spillover since it is unclear what proportion of viral evolution occurred in unsampled humans prior to transmission to deer and what occurred while circulating within deer.

For the delta variant (Fig. 4), 5 deer genomes annotated as delta, and all 6 spike sequences showed patterns also consistent with delta (Table S3). Two deer genomes were assigned to the AY.103 clade, 2 genomes to AY.88, and 1 to AY.5. The delta lineages from deer were not as diverged from related human sequences as the alpha lineages, consistent with more recent introductions and less long-term circulation in deer. This is as expected since delta became widespread in the US only shortly before the time of sampling. Molecular clock analysis suggested that the closely related AY.103 pair of genomes likely entered the deer population as early as early September 2021 (95% CrI: 8/8/2021 to 10/4/2021); the AY.5 lineage entered as early as mid-August 2021 (95% CrI: 7/31/2021 to 8/27/2021); and the AY.88 pair entered deer as early as mid-October 2021 (95% CrI: 9/30/2021 to 10/31/2021) (Fig. S2). For the delta cases, the proposed jumps from deer to humans likely occurred during the height of the delta wave. The 6 samples sequenced as single spike amplicons also displayed mutations consistent with delta variants and were collected at times and locations similar to the fully sequenced delta variant genomes from deer. The small size of these amplicons prevents assignment to clade and identification of transmission events but confirms the extensive presence of delta in deer.

## DISCUSSION

These data support 5 independent transmissions of SARS-CoV-2 from humans to deer in the samples from Pennsylvania analyzed. These include one jump each for the 2 alpha sequences, and 3 for the delta sequences (Fig. S2). Other scenarios are also possible, involving more independent jumps and convergent evolution in deer, or fewer jumps and divergent evolution along trajectories matching trajectories in humans.

Mechanisms of infection and transmission in deer are incompletely understood. Studies of experimental infections show efficient transmission between deer, potentially involving close interactions such as touching noses and grooming (25). The mechanism of transmission from humans to deer remains obscure, though evidence here of multiple transmission events suggests it is not a rare event.

This study has several limitations. The sample size is modest, and whole genome sequence acquisition was limited by viral RNA concentrations in samples. Our sample size limits the interpretation of epidemiological findings (e.g., differences in infection rates between sample types), though does not undermine the primary findings of this work (e.g., divergence of the alpha lineage in deer, and evidence for multiple spillovers). The background human data is incomplete, with only an estimated 1.7% percent of all human cases in PA subjected to viral whole genome sequencing, limiting our ability to identify and correctly time spillover and spillback events. Each evolutionary analysis of sequences requires assumptions on the structures of background populations that are not fully investigated. In addition, we were not able to obtain serum samples from the deer studied and so could not investigate immune responses.

In summary, we report a survey of SARS-CoV-2 in 123 deer in Pennsylvania over the fall-winter of 2021, which showed 16% of the deer to be positive. Prior surveys carried out over the fall and winter of 2020 also showed high point prevalence of infection in Iowa (33%) (17) and Ohio (36%) (11), and later extensive infection at other locations (20, 26–28). We report the first examples of the alpha and delta lineages in wild white-tail deer and identify five likely independent spillovers from humans to deer among seven fully sequenced genomes. Given that there are estimated to be 30 deer per square mile in PA, and over a million deer total, this suggests an enormous number of spillovers and infected deer in the state (29, 30). Our findings of alpha persistence in deer after replacement of alpha by delta in humans, and the divergence seen between our deer and human alpha genomes, are all consistent with long-term persistence and spread of the alpha variant in deer. Yet, there is no evidence for spillback of the deer lineages identified here into humans; ongoing efforts to characterize human and deer SARS-CoV-2 lineages are valuable to maintain surveillance for such events.

## MATERIALS AND METHODS

### Collection of samples from white-tailed deer.

Samples were collected from hunter-harvested deer and injured deer that were euthanized by state game wardens. Nasal swabs were taken within hours of death, placed in phosphate-buffered saline (PBS) and stored in commercial refrigerators in field offices. Samples were shipped to the University of Pennsylvania within 1 week of collection and stored at –80°C until RNA extraction. Fisher’s Exact tests were performed using R Statistical software (v4.0.5) (31) (R Core Development Team 2021) to test for differences in proportions of virus positive and negative deer by sex, age, and cause of death (i.e., hunter-harvested, road-killed, or all other causes of death).

### RNA extraction and detection of SARS-CoV-2 RNA by RT-qPCR.

Nucleic acid was extracted with a QIAamp viral RNA minikit (Qiagen) according to the manufacturer’s instructions. The presence of SARS-CoV-2 RNA was assessed by a RT-qPCR targeting 2 regions of the viral nucleocapsid gene as described previously (32).

### SARS-CoV-2 whole genome sequencing.

Viral genomes were sequenced using the POLAR protocol (33). The sample RNA (5 μL) was mixed with Random Hexamers (0.5 μL of 50 μM, Thermo Fisher), dNTPs Mix (0.5 μL of 10 mM each, Thermo Fisher), and nuclease-free water (1 μL). This mixture was incubated (5 min at 65°C). Subsequently, reverse transcription was performed adding 6.5 μL from the previous reaction to SuperScript III Reverse Transcriptase (0.5 μL, Thermo Fisher), 5X First-Strand Buffer (2 μL, Thermo Fisher), DTT (0.5 μL of 0.1M, Thermo Fisher), and RNaseOut (0.5 μL, Thermo Fisher). This mixture was incubated (50 min at 42°C, then 10 min at 70°C). For cDNA amplification, the previous mixture (2.5 μL) was added to Q5 Hot Start DNA polymerase (0.25 μL, NEB), 5X Q5 Reaction Buffer (5 μL, NEB), dNTPs mix (0.5 μL of 10 mM each, NEB), and pooled Artic-ncov2019 v4 primers (reaction set 1 used 4.0 μL of pooled primer set 1, reaction set 2 used 3.98 μL of pooled primer set 2, IDT) and water (volume reaction brought to 25 μL). The samples were brought to 98°C for 30 s then cycled from 98°C for 15 s to 65°C for 5 min for 25 cycles, followed by 98°C for 15 s and 65°C for 5 min. The 2 reactions (one for each primer set) were pooled together. Tagmentation was completed with the Nextera XT Library Preparation Kit (Illumina). IDT for Illumina DNA/RNA UD Indexes were used for barcoding (Illumina). Quantification of DNA was performed using Quant-iT PicoGreen dsDNA quantitation assay kit (Invitrogen). The pooled libraries were quantified using the Qubit1X dsDNA HS assay kit (Invitrogen) and sequenced using the Illumina NextSeq platform.

### SARS-CoV-2 spike amplicon sequencing.

Amplicon sequencing was carried out using nested PCR (34) to target the spike coding region. Samples with genomic viral load deemed too low for whole genome sequencing, or samples that failed whole genome sequencing, underwent nested PCR targeting the spike’s receptor binding domain sequence. Primers used are provided in Table S7. PCR 1 includes reverse transcription and initial DNA amplification using the Superscript IV One-Step RT-PCR System (Thermo Fisher Scientific,12594100). The 25 μL reaction consists of 5 μL of extracted viral RNA (extracted as described in the whole genome sequencing section), 0.25 μL of Superscript IV, 12.5 μL of 2X Platinum SuperFi RT-PCR mix, 1.25 μL of each PCR 1 primers (10 μM), 4.75 μL molecular grade water. The cycle conditions are as follows: 25°C for 2 min, 50°C for 20 min, 95°C for 2 min, 25 cycles of amplification (95°C for 2 min, 55°C for 30 s, 70°C for 1 min). PCR 2 is a nested PCR and addition of sequencing adapters for the amplicon product from reaction 1. The 25 μL mixture contains 5 μL of amplicon product from reaction 1, 12.5 μL 2x Q5 hot start master mix (NEB, M0494S), 0.5 μL of 10 mM dNTPs (NEB, N0447S), 1.25 μL of each PCR 2 primers (10 μM), and 7.5 μL water. The conditions are as follows: 95°C for 2 min, 20 cycles of amplification (95°C for 15 s, 55°C for 30 s, 72°C for 1 min). PCR 3 adds the unique dual indexes and clustering adapters. The 50 μL reaction contains 25 μL of amplicon product from PCR 2, 0.5 μL of Phusion polymerase (NEB, M0530L), 10 μL of 5x Phusion buffer, 1 μL of 10 mM dNTPs (NEB, N0447S), 5 μL of IDT for Illumina DNA/RNA UD Indexes set A (Illumina, 20027213), and 8.5 μL of water. The reaction conditions are as follows: 98°C for 3 min, 7 cycles of amplification (95°C for 15 s, 50°C for 30 s, 72°C for 30 s), and 72°C for 7 min. 10 μL of the barcoded PCR 3 mixture was pooled together, AMPure purified, and quantified by Qubit1X dsDNA and TAPE station before being sequenced on an Illumina MiSeq instrument using a 600 cycle v3 standard flow cell 290/10/10/290 protocol (Illumina, MS-102-3003). After sequencing, BWA v0.7.17-r1188 was used to generate bam files, Samtools v1.10 (using htslib 1.10.2-3) was used to filter reads for lengths between 280 and 300 bp, filter reads by quality using a PHRED threshold of 30, sort the reads, index the reads, and generate a pileup. Samples must meet a minimum mapping quality PHRED score of 30, have 95% 50-fold coverage for the 550 bp amplicon target to pass additional filtering. Substitutions were called with a greater than 0.67 allele frequency for a given position.

10.1128/mbio.02101-22.7 TABLE S7 Key materials and resources. Download Table S7, PDF file, 0.4 MB. Copyright © 2022 Marques et al. 2022 Marques et al. https://creativecommons.org/licenses/by/4.0/ This content is distributed under the terms of the Creative Commons Attribution 4.0 International license.

### Sequence data processing.

Sequence data were processed as previously described (23). BWA aligner tool (v0.7.17) was used to align viral sequences to the Wuhan reference sequence (NC_045512.2) (35). Samtools package (v1.10) was used to filter alignments (36). Variants were called using Pangolin lineage software (3.1.17 with the PangoLEARN 2021-12-06 release) (22, 37). NVSL’s vSNP pipeline was also run on deer samples and results compared to those generated by our published pipeline; findings from both platforms deviated only for thresholds used to call low abundance substitutions. All variant calls remained the same between platforms.

### Host sequence analysis.

The proportion of host sequences was inferred using a sampling of raw reads from all samples on any sequencing batch performed with deer specimen. 1,000 raw reads for each sample were blasted (38–40) against a database constructed of a SARS-CoV-2 genome (NC_045512.2), human genome (GCF_000001405.39), and white-tailed deer genome (GCF_002102435.1). Reads were tallied for each genome to which they were closely matched.

### Mutation analysis.

One hundred eight deer-derived SARS-CoV-2 genomes were downloaded from GISAID and compared with the 7 deer sequenced in this publication and the human data set (Tables S4 and S5). The previously published genomes were downloaded on 12/29/2021. Mutations were called in reference to the Wuhan strain (NC_045512.2).

### Bayesian analysis of county proportions.

To account for the variable sampling between counties and potential similarities between neighboring counties, we estimated the underlying proportion of deer testing positive within each county of the *m* counties using a Bayesian conditional autoregressive model. The number of positive tests, *y_i_*, out of *n_i_* total tests within each county *i* was modeled as:

$$y_{i}\;\sim \;\text{Binomial}(p_{i},n_{i})$$

where *p_i_* is the proportion of deer expected to test positive in that county and:

$$p_{i}={\text{logit}}^{-1}(\alpha +\beta_{i})$$

Here, *α* represents the average proportion positive for a county and the vector of differences from this average for each county, *β*, is distributed as a multivariate normal:

$$\beta \;\sim \;{\text{Normal}}_{\text{prec}}(0,\frac{1}{\sigma }(D-\theta A))$$

where *D* is a m×m matrix with 0s on the off diagonal and the diagonal element on each row *i*, *D_i,i_*, equal to the number of counties that are adjacent to county *i*, *Α* is a m×m adjacency matrix with element *Α*_i,j_ is 1 if county *i* neighbors county *j* and 0 otherwise and the diagonal set to 0 and ${\mathrm{Normal}}_{\mathrm{prec}}(x,y)$ is a multivariate normal distribution with means *x* and precision matrix *y*. For priors, θ was given a uniform prior between 0 and 1, σ ~ Gamma(1,1) and α ~ Normal(-2,10).

Posterior probability distributions were estimated using Markov chain Monte Carlo sampling using Stan v2.21.0 (41).

County adjacency data was obtained from the US Census Bureau (https://www.census.gov/geographies/reference-files/2010/geo/county-adjacency.html).

### Time-scale Bayesian maximum clade credibility tree.

A phylogenetic approach using a time scale Bayesian maximum clade credibility (MCC) tree was used to estimate the introduction of SARS-CoV-2 into PA white-tailed deer. We subsampled genomes based on nearest human neighbor to deer sequences using NCBI’s global nucleotide data set consisting of 943,071 complete SARS-CoV-2 genomes. To determine the nearest human neighbors, a BLASTn search was used. If a deer’s nearest hits included another deer, then both were analyzed in the same tree. Sequences without exact dates of collection were excluded. Sample sizes were chosen as the 100 nearest neighbor whole genome sequence isolates from humans. Each data set was aligned using NextClade with Wuhan-Hu-1 as a reference (NC_045512.2). A Markov chain Monte Carlo (MCMC) method was used to generate time-scales Bayesian molecular clocks using BEAST v.1.10.4 (42). Several parameters were assessed using path sampling and stepping-stone sampling of marginal likelihood estimation (Table S6). The best performing settings were a general time reversible substitution model with gamma-distributed rate variation among sites (Yang96) (43, 44) with an uncorrelated relaxed lognormal clock to allow for branch-specific variation in evolutionary rates (45–47) and a Bayesian Skyline tree prior with a group size of 10 (48). A previously published SARS-CoV-2 paper examining infection of white-tailed deer also converged on similar parameters (11) and similar parameters performed well in a comparison of potential models of SARS-CoV-2 evolution (49). MCMC sampling was run for 100 million iterations with subsampling every thousand iterations and 10 million iterations discarded as burn-in. The BEAGLE 3 library was used to improve computational performance (50). Tracer v.1.7.1 was used to visually assess convergence, TreeAnnotator v.1.10.4 (42) to summarize the MCC tree and FigTree v.1.4.4 (51) to visualize the tree. All tree analyses were repeated 3 times independently and visually checked for convergence and all effective sample sizes were confirmed to be greater than 200 using Tracer v1.7.2 (52). Maximum-likelihood trees were generated by IQ-TREE v1.6.12 (53) using the samples selected for the BEAST analysis and the root-to-tip genetic distances were compared to collection dates using a rooting maximizing correlation with TempEst v1.5.3 (54) to assess sample selection and evolutionary rates (Fig. S3).

10.1128/mbio.02101-22.10 FIG S3 Root-to-tip plot for time resolved phylogenies compare deer and human isolates. Deer samples are highlighted. Plots are shown for individual lineages. Accessions for included samples can be found in Table S5. Download FIG S3, PDF file, 0.9 MB. Copyright © 2022 Marques et al. 2022 Marques et al. https://creativecommons.org/licenses/by/4.0/ This content is distributed under the terms of the Creative Commons Attribution 4.0 International license.

### Human subjects.

Human sequences newly determined here were collected as follows. For most samples, the University of Pennsylvania Institutional Review Board (IRB) reviewed the research protocol and deemed the limited data elements extracted to be exempt from human subject research per 45 CFR 46.104, category 4 (IRB #848605). For hospitalized subjects, following informed consent (IRB protocol #823392), patients were sampled by collection of saliva, oropharyngeal and/or nasopharyngeal swabs, or endotracheal aspirates if intubated, as previously described (23). Further samples were collected from asymptomatic subjects detected in a screening program at the Perelman School of Medicine at the University of Pennsylvania and symptomatic subjects tested throughout the PennMedicine clinical network under IRB protocols #843565 and #848608. Human samples were sequenced as described for deer samples.

### Phylogenetic analysis.

Deer and human viral sequences are available at GenBank (Table S2 and S5). Samples used include i) deer viral sequences of the same variant, ii) 100 nearest human viral sequences from a global search, and iii) all human viral sequences of the same variant from our local Delaware River Valley data set. NextClade was used to align sequences to the Wuhan reference (53). IQ-TREE (v1.6.12) was used to infer a tree using maximum-likelihood methods using 1,000 bootstrap replicates (55). Visualization of the inferred tree was performed using iTOL v6.

### Materials.

Key materials and resources are compiled in Table S7.

### Data availability.

Sequence accession numbers for deer-derived SARS-CoV-2 genomes can be found in Table S2 (OM570187-OM570193 & ON350842-ON350847). Accession numbers for human SARS-CoV-2 genomes can be found in Table S5. Sequence processing and analysis code are deposited at https://doi.org/10.5281/zenodo.4046252 and https://doi.org/10.5281/zenodo.6842232. BEAST/BEAUti xml files used for the Bayesian maximum credibility trees are available at https://zenodo.org/record/6761298#.YrnKZXbMKUk.
