# Supplementary material for: Multiple Introductions of SARS-CoV-2 Alpha and Delta Variants into White-Tailed Deer in Pennsylvania
Source: mBio. 2022 Aug 24;13(5):e02101-22. doi: 10.1128/mbio.02101-22 (PMC9600874; doi:10.1128/mbio.02101-22)
Supplement: TABLE S1 [file mbio.02101-22-s0001.pdf]

Table S1. SARS-CoV-2 prevalence estimates stratified by sex, age, sampling region, and cause of death

	Category	Total	Positives	Mean	Lower 95% Interval	Upper 95% Interval
**Sex**	Male	74	14	18.9%	11.1%	30.0%
	Female	49	6	12.2%	5.1%	25.5%
**Age**	Fawn	18	1	5.6%	0.3%	29.4%
	Yearling	22	1	4.5%	0.2%	24.9%
	Adult	81	17	21.0%	13.0%	31.7%
	Unknown Age	2	1	50.0%	9.5%	90.5%
**Region**	NER	30	11	36.7%	20.5%	56.1%
	NCR	5	0	0.0%	0.0%	53.7%
	NWR	18	4	22.2%	7.4%	48.1%
	SER	31	1	3.2%	0.2%	18.5%
	SCR	32	3	9.4%	2.5%	26.2%
	SWR	5	0	0.0%	0.0%	53.7%
**Cause of Death**	Hunter Harvested	79	12	15.2%	8.4%	25.4%
	Road Kill	24	7	29.2%	13.4%	51.2%
	Unkown Cause of Death	20	1	5.0%	0.3%	26.9%
