# Supplementary figures and images for: Multiple Introductions of SARS-CoV-2 Alpha and Delta Variants into White-Tailed Deer in Pennsylvania
Source: mBio. 2022 Aug 24;13(5):e02101-22. doi: 10.1128/mbio.02101-22 (PMC9600874; doi:10.1128/mbio.02101-22)

**Figure S1**

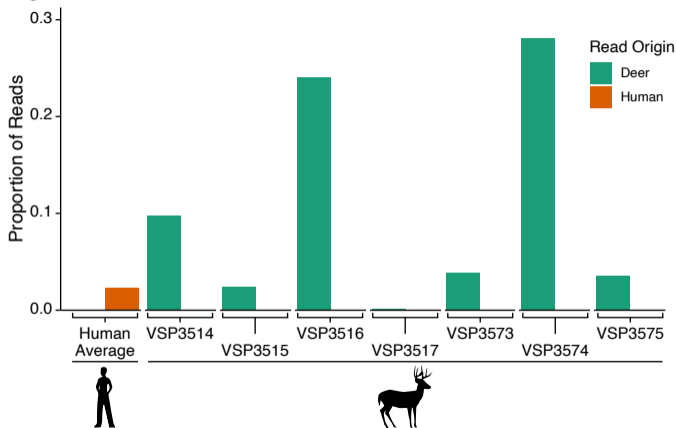

Supplement: FIG S1 [file mbio.02101-22-s0008.pdf]

Figure S2

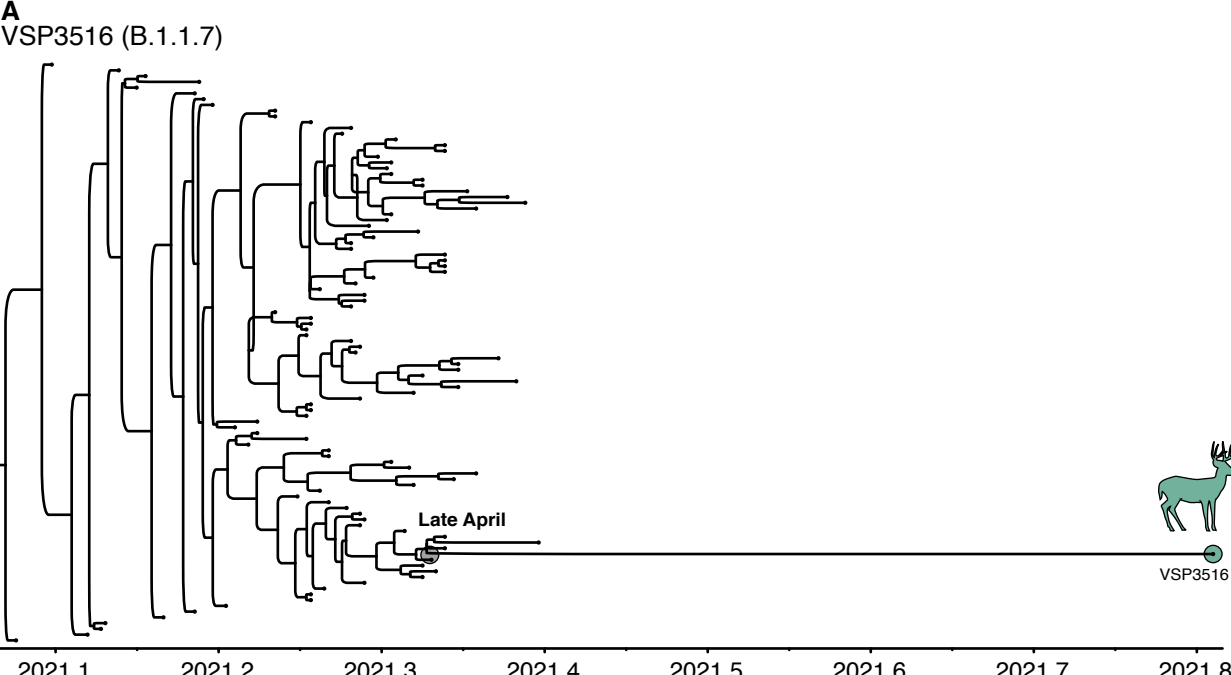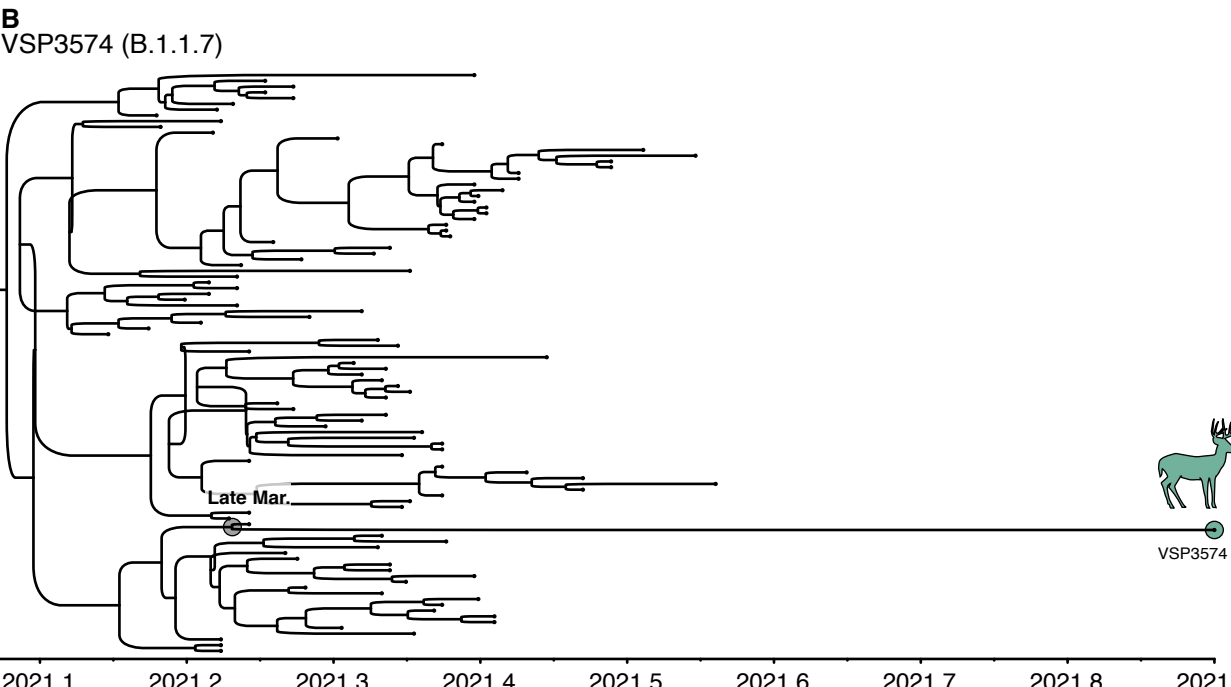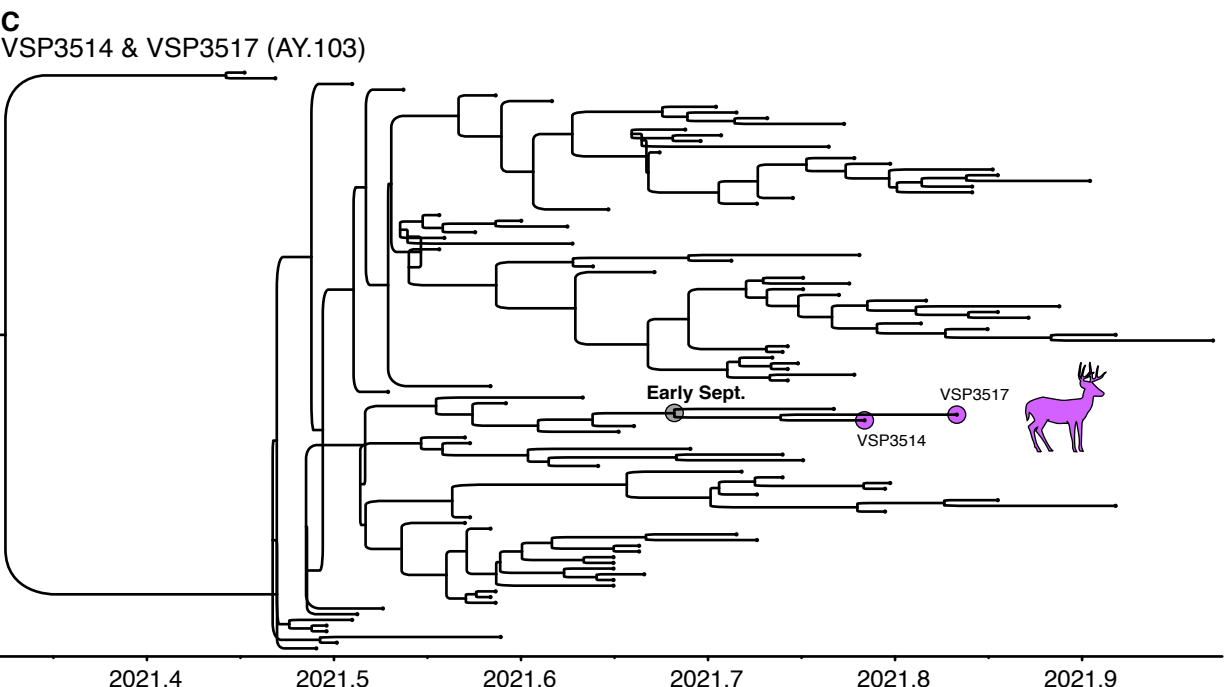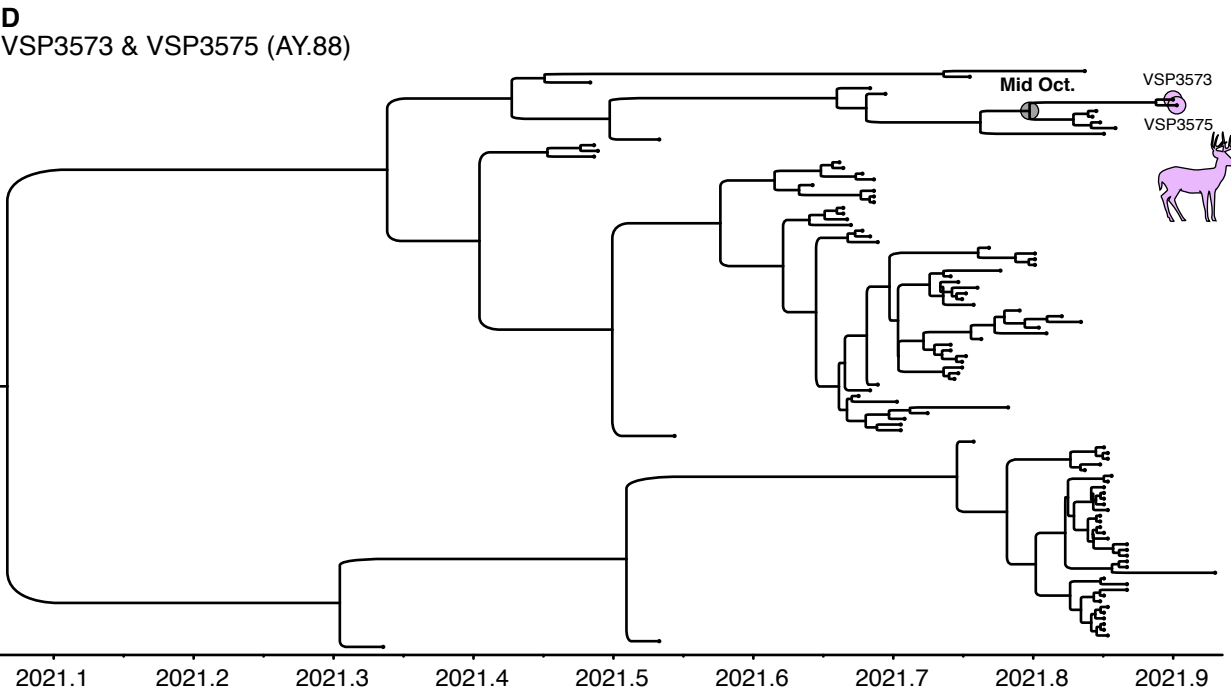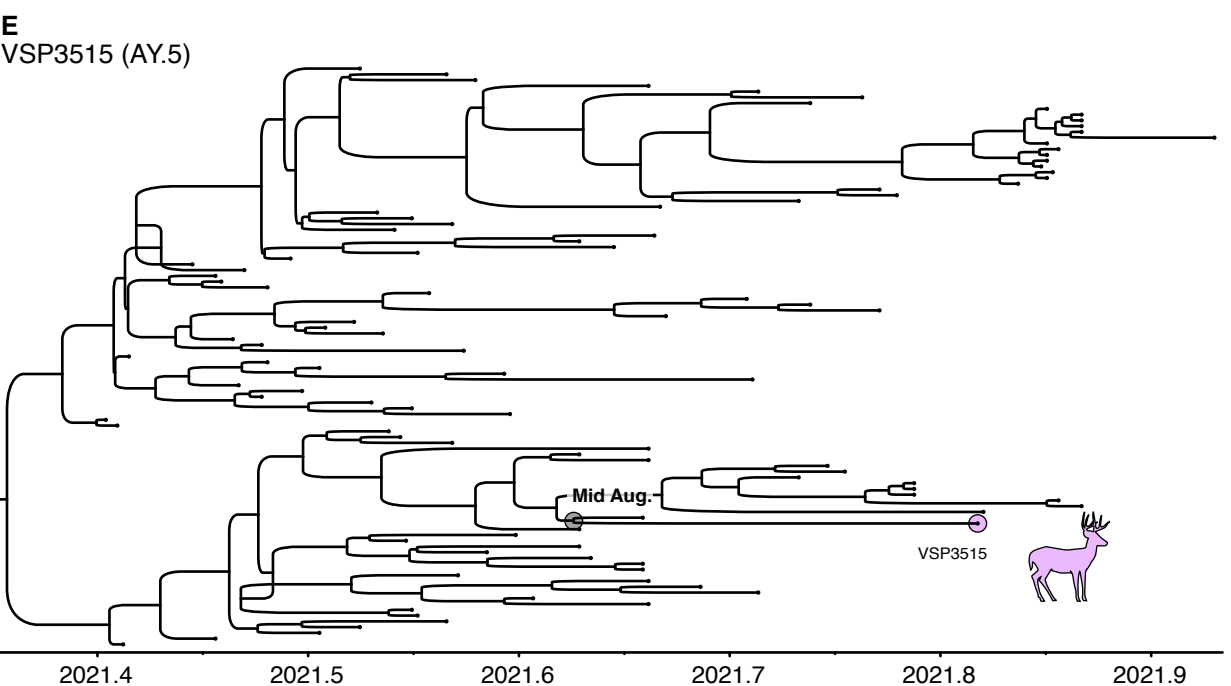

Supplement: FIG S2 [file mbio.02101-22-s0009.pdf]

**Figure S3**

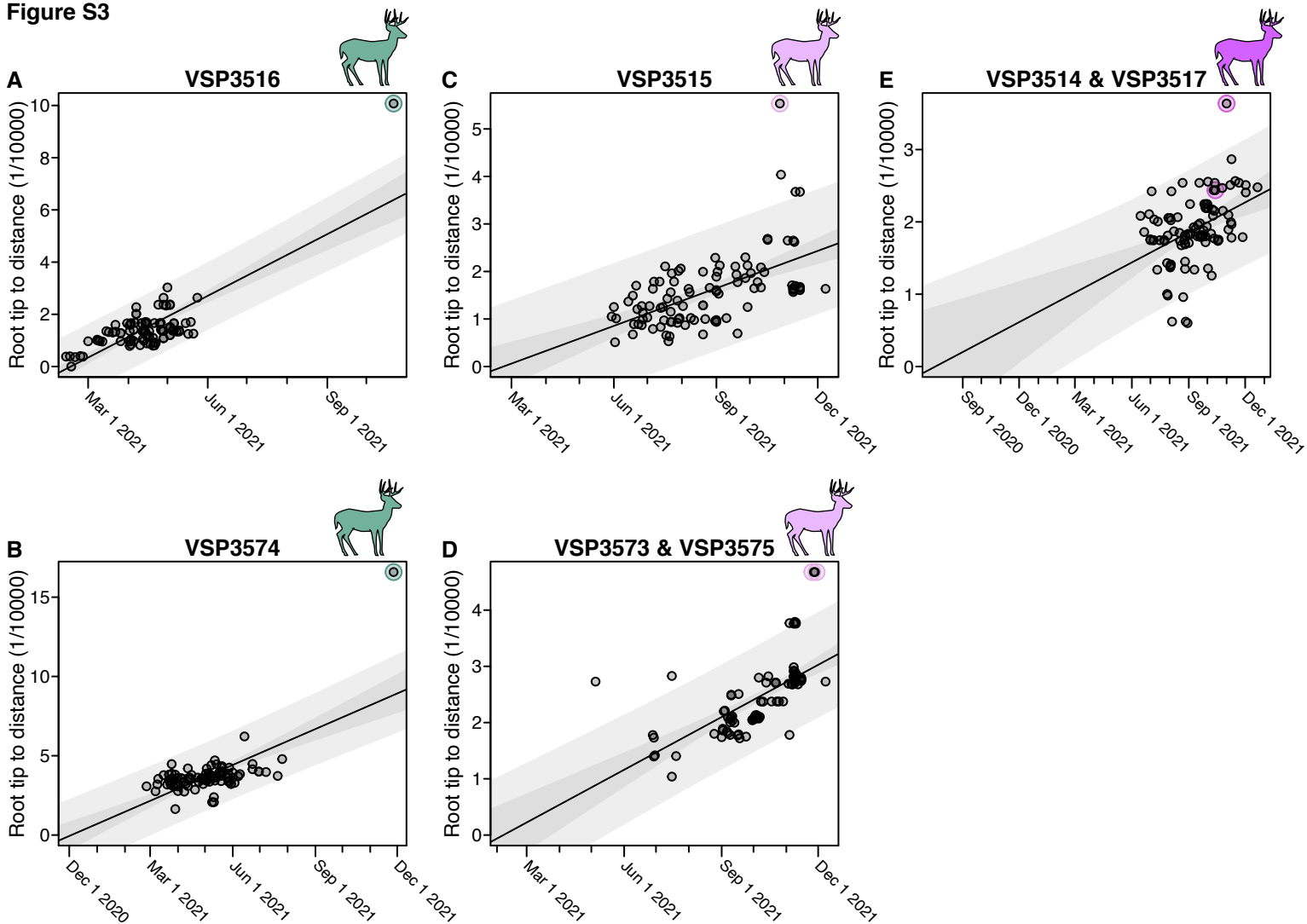

Supplement: FIG S3 [file mbio.02101-22-s0010.pdf]
