# Supplementary material for: Multiple Introductions of SARS-CoV-2 Alpha and Delta Variants into White-Tailed Deer in Pennsylvania
Source: mBio. 2022 Aug 24;13(5):e02101-22. doi: 10.1128/mbio.02101-22 (PMC9600874; doi:10.1128/mbio.02101-22)
Supplement: TABLE S6 [file mbio.02101-22-s0006.pdf]

Table S6. Parameter tuning for time-scale Bayesian maximum clade credibility tree					
**Clock Model**	**Tree coalescent**	**Substitution model**	**Stepping-stone sampling**	**Path sampling**	**Optimal?**
Strict Clock	Constant Size	Yang96	-46552.73	-46552.62	No
UCLN	Exponential	SRD06	-46850.92	-46850.95	No
UCLN	Exponential	Default HKY	-46880.58	-46880.25	No
UCLN	Exponential	SRD06	-46849.74	-46849.09	No
UCLN	Bayesian Skyline	SRD06	-46844.39	-46843.45	No
UCLN	Bayesian Skyline	Yang96	-46484.31	-46483.49	Yes
