# Supplementary material for: Multiple Introductions of SARS-CoV-2 Alpha and Delta Variants into White-Tailed Deer in Pennsylvania
Source: mBio. 2022 Aug 24;13(5):e02101-22. doi: 10.1128/mbio.02101-22 (PMC9600874; doi:10.1128/mbio.02101-22)
Supplement: TABLE S7 [file mbio.02101-22-s0007.pdf]

Table S7. Key materials and resources

Reagent or Resource	Source	Identifier
QIAmp 96 Viral RNA Kit	Qiagen, Hilden, Germany	5262
SupperScript III RT	Thermo Fisher Scientific, Waltham, USA	56575
SS III First Strand 5x Buffer	Thermo Fisher Scientific, Waltham, USA	Y02321
Random Hexamers	Thermo Fisher Scientific, Waltham, USA	51709
Dithiothreitol	Thermo Fisher Scientific, Waltham, USA	Y00122
Molecular Grade Water	Thermo Fisher Scientific, Waltham, USA	Y01138
Deoxynucleotide Mix	New England Biolabs, Ipswich, USA	N0447S
ARTIC Primer Pool 1	Integrated DNA Technologies, Coralville, USA	100006786
ARTIC Primer Pool 2	Integrated DNA Technologies, Coralville, USA	100006787
Q5 Hot Start Polymerase	New England Biolabs, Ipswich, USA	M0493L
Q5 5x Reaction Buffer	New England Biolabs, Ipswich, USA	B90275
AMPure XP	Beckman Coulter, Brea, USA	A63882
Qubit™ 1X dsDNA Kit	Invitrogen Corp., Waltham, USA	Q33230
Quant-iT PicoGreen Kit	Invitrogen Corp., Waltham, USA	P7589
IDT for Illumina DNA/RNA UD Indexes A	Illumina Inc., San Diego, USA	20027213
IDT for Illumina DNA/RNA UD Indexes B	Illumina Inc., San Diego, USA	20027214
IDT for Illumina DNA/RNA UD Indexes C	Illumina Inc., San Diego, USA	20027215
IDT for Illumina DNA/RNA UD Indexes D	Illumina Inc., San Diego, USA	20027216
Nextera XT DNA Library Preparation Kit	Illumina Inc., San Diego, USA	FC-131-1096
NextSeq 500/550 Mid Output Kit v2.5 (150 Cycles)	Illumina Inc., San Diego, USA	20024904
QiAmp Viral RNA mini kit	Qiagen, Hilden, Germany	52906
VetMax Xeno IPC RNA	Thermo Fisher Scientific, Waltham, USA	A29763
TaqPath 1 Step RT-qPCR master mix	Thermo Fisher Scientific, Waltham, USA	A15299
VetMax Xeno VIC Assay	Thermo Fisher Scientific, Waltham, USA	A29765
2019-nCov CDC EUA Kit	Integrated DNA Technologies, Coralville, USA	10006713
MiSeq Reagent Kit v3	Illumina Inc., San Diego, USA	MS-102-3003
Superscript IV One-Step RT-PCR System	Thermo Fisher Scientific, Waltham, USA	12594100
Phusion polymerase	New England Biolabs, Ipswich, USA	M0530L
2x Q5 hot start master mix	New England Biolabs, Ipswich, USA	M0494S

Nested Spike PCR Sequencing Primers

Description	Sequence	Details
PCR 1 Forward	CTGCTTTACTAATGTCTATGCAGATTC	Targets SARS-CoV-2 spike RBD
PCR 1 Reverse	TCCTGATAAAGAACAGCAACCT	Targets SARS-CoV-2 spike RBD
PCR 2 Forward	tcgtcggcagcgtcagatgtgtataagagacagGTGATGAAGTCAGACAAATCGC	sequence adapter | SPIKE NESTED PRIMER
PCR 2 Reverse	gtctcgtgggctcggagatgtgtataagagacagATGTCAAGAATCTCAAGTGTCTG	sequence adapter | SPIKE NESTED PRIMER
